# Supplementary figures and images for: Comparative study of the unbinding process of some HTLV-1 protease inhibitors using unbiased molecular dynamics simulations
Source: PLoS One. 2022 Jul 14;17(7):e0263200. doi: 10.1371/journal.pone.0263200 (PMC9282663; doi:10.1371/journal.pone.0263200)

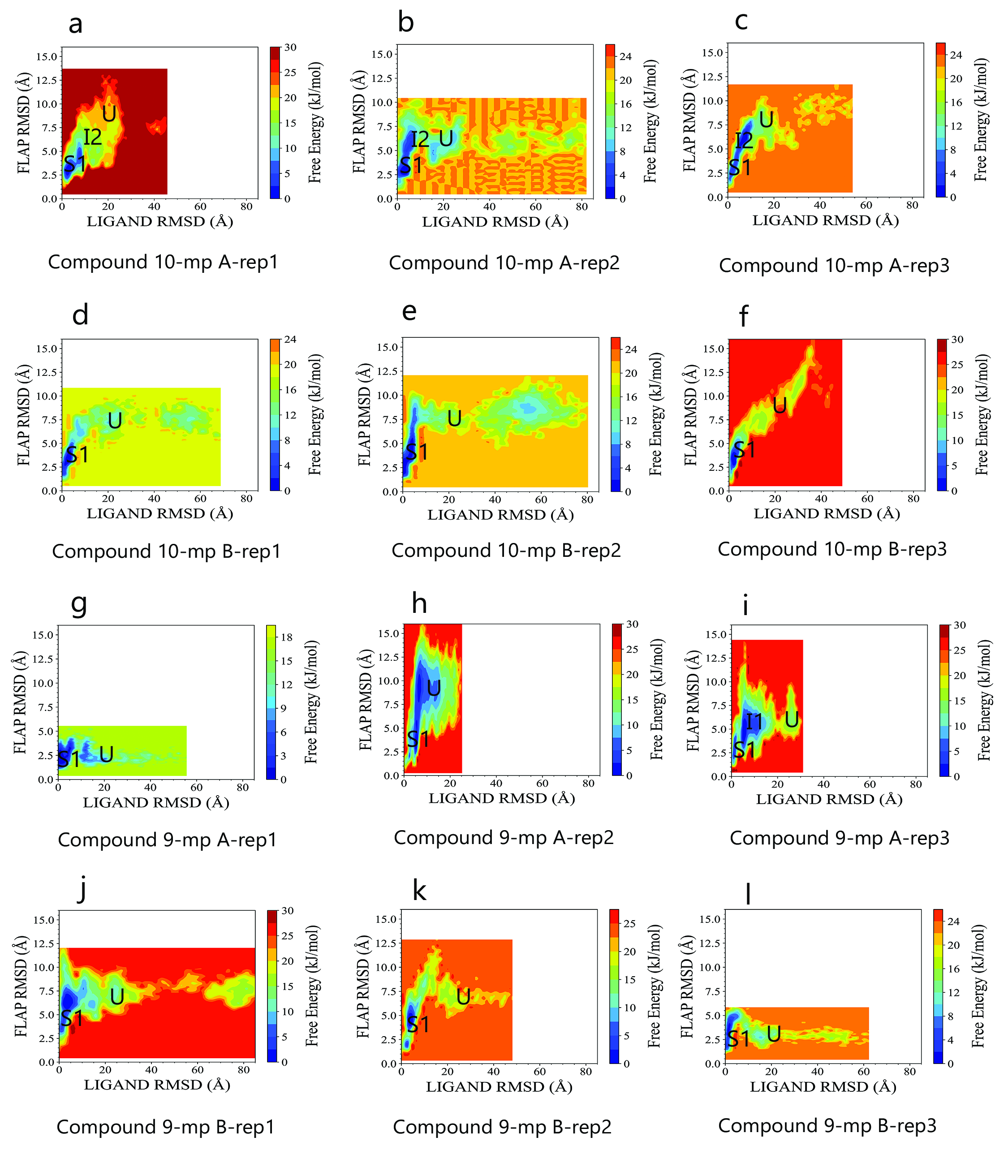

Supplement: S1 Fig — (TIF) [file pone.0263200.s001.tif]

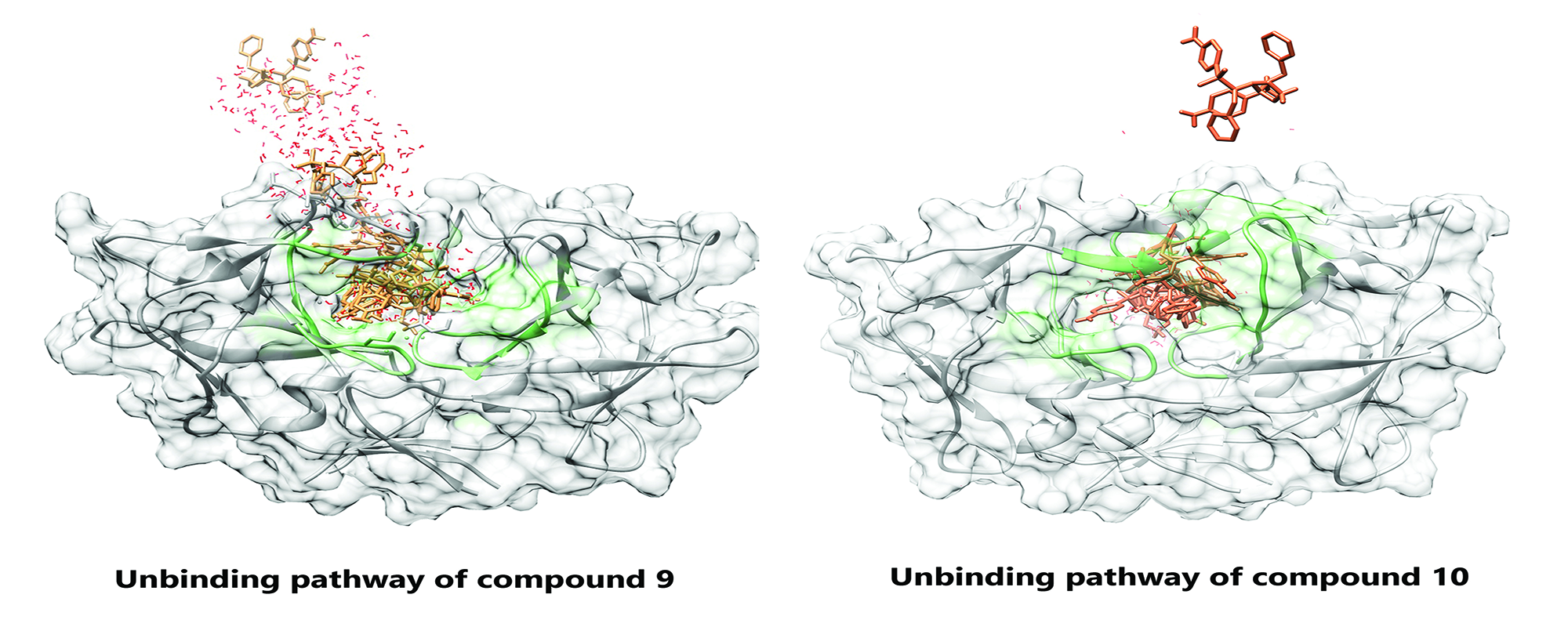

Supplement: S2 Fig — (TIF) [file pone.0263200.s002.tif]
