# Supplementary material for: Comparative study of the unbinding process of some HTLV-1 protease inhibitors using unbiased molecular dynamics simulations
Source: PLoS One. 2022 Jul 14;17(7):e0263200. doi: 10.1371/journal.pone.0263200 (PMC9282663; doi:10.1371/journal.pone.0263200)
Supplement: Attachment — Submitted filename: Reviewers feedback.docx [file pone.0263200.s003.docx]

In this paper, Tiyoula and co-workers studied the unbinding process of Human T-cell leukemia virus with the help of molecular dynamics simulation. The study is interesting, and the analysis are detailed but rigorous changes are required to make it publishable. Some important points are depicted below-

1. After lots of simulations and analyses, it is still unclear that which states of protonation plays the deciding role there. It would be better if the authors are critical on this point because it is one of the most important viewpoints of this work.

2. Authors in many places discussed about pi-cation and pi-anion interaction (e.g., His66 interacts with aniline in cation-pi interaction, Asp36 forming pi-anion interaction with nitrobenzene etc.). It is important to note that the energetics of such interactions are very important. (For example, pi-anion interactions have energetics in the range around 20 to 50 kj/mol whereas pi-cation interactions have energetics in the range of -10 to -20 kj/mol). The authors should check the energetics of such interactions here or at least cite relevant papers where people have reported the energetics of these particular system.

3. Authors should rigorously work on the figure qualities. Those are not good at publication format presently. For example, Figure 13, c, d, e. Note that, the labellings are very bold and not as per the mark of publication. Also, in 13a, the x-axis labellings are very closely spaced (10,20,30,40….80) makes it looks worse. They should scrutinize the figure qualities and font labelling throughout in-depth.

4. In Figure 10 caption (where they emphasize the details of compound 9 unbinding pathway), just before the last line, they mentioned “k, l, 3”, What is “3” here? I did not find anything such and believe “3” should be replace by “m”. Such things they should check thoroughly.

5. It is better to follow a uniform free energy bar throughout which is currently missing. For example, Figure 7, c, d, e. Their energy bars are not uniform (For c, it is 0 to 16.90 and it is different for d and e.). The authors should follow uniform energy bar for all, otherwise the comparison is not at all logical.

6. The authors mention that “For our purpose, we had various short and long-time-scale simulations”. I understand they chose based on the unbinding time scales, still did the authors performed any convergence tests or something like that, to choose time scales in this study?

7. Figure 5, for the three replica’s they chose three different time scales, that should be addressed properly. What happened if for Figure 5b and 5c, they did 4000 ns simulations like Figure 5a? Then, how the distance plot would look like? Since, they are doing unbiased simulations, these questions need to be answered. Also, it is visually more sound to have similar time-scales for different replica’s.

8. Figure 3g, take care of the x axis label. It has been cut-off (120 ns is cut-off there). Same goes for Figure 8c. Also, The TOC (as well as TOC fonts) is not looking good as per the journal standard.

9. The article is somewhat looks to be less referenced. For an example, they should cite some important references regarding the interactions such as the review articles sastry et al doi: 10.1021/cr300222d and doi: 10.1021/jp900013e, and some others, for example <https://doi.org/10.1021/ct200569x>, <https://doi.org/10.1021/acs.jpcb.7b01736> and <https://doi.org/10.1021/acs.jpcb.9b06343> etc.
