# Supplementary material for: Comparative study of the unbinding process of some HTLV-1 protease inhibitors using unbiased molecular dynamics simulations
Source: PLoS One. 2022 Jul 14;17(7):e0263200. doi: 10.1371/journal.pone.0263200 (PMC9282663; doi:10.1371/journal.pone.0263200)
Supplement: Attachment — Submitted filename: Response to Reviewers.docx [file pone.0263200.s004.docx]

**Dear Prof. Sandipan Chakraborty;**

I am pleased to mail you the revised article titled **"Comparative study of the unbinding process of some HTLV-1 protease inhibitors using Unbiased Molecular Dynamics simulations"**. All comments have been answered upon reviewers' recommendations, and the corrections were made in manuscript # PONE-D-22-00753. It is our pleasure to hear your feedback and any other suggestions relevant to the paper.

**Sincerely,**

**Hassan Aryapour**

**Reviewer #1:**

**Comment #1:**

The abstract is very poorly written. Rewrite the abstract according to findings.

**Answer #1:**

The abstract section was improved and rewritten.

**Comment #2:**

Clearly define the aim and objectives of the study in the last paragraph of the introduction section.

**Answer #2:**

The requested information was added in the last paragraph of the introduction section (lines 100-104)

**Comment #3:**

In the Introduction section the author should refer to the research paper and comment on recent in-silico techniques. It will be good information for the readers. I would like to recommend several papers, among many others, providing further explanation on this topic: PMID: 27194485 PMID: 33749525 PMID: 32448055 PMID: 31980008 PMID: 33065246 PMID: 32447145 PMID: 34717229

**Answer #3:**

Some of your mentioned references were added as an example of some in silico methods (lines 72-79)

**Comment #4:**

Authors have advised redrawing all the interaction energy graphs in reverse order.

**Answer #4:**

In this study, we examined unbinding pathways of mentioned inhibitors, so at first, the inhibitors were in a binding position with the highest amount of interaction energies. During the simulation, the weakening of protein-ligand interactions occurred. The inhibitors started to leave the binding site and eventually full unbind occured when the total amount of protein-ligand interaction energies reached zero. So all the interactions energies plots' trend is correlated with the unbinding process from the bound state to the unbound state. This kind of analysis also used in the other studies:

<https://doi.org/10.1371/journal.pone.0263251>,https://doi.org/10.1371/journal.pone.0257916 https://doi.org/10.1371/journal.pone.0251910

**Comment #5:**

The interaction energy values appear to be much larger than those of typical small molecule inhibitors. I think the interaction energy is insufficient to determine the unbinding mode of the ligand accurately. Is there a possibility of overestimation such as incorrectness of reweighing or insufficient sampling of the unbound state?

**Answer #5:**

Total interaction energies plots are obtained from the sum of the coulomb and Lennard-jones contribution values, so the total energies are high. To better understand the complementary analysis, we calculate the average of the most important interaction energies (the amount of these plots is not much as the Total interaction energies plots).

Investigating the energy plots was not the only analysis for determining unbinding processes. We also used the free energy landscape plots obtained using "gmx sham", based on the ligand and protein RMSD values. Eventually, all preliminary information was investigated with trajectory files to advanced details obtain.

**Comment #6:**

The authors have provided insufficient data. Authors have advised to perform binding studies first before exploring the unbinding.

**Answer #6:**

Along with investigating the unbinding pathways of these two inhibitors we also investigated the binding pathways of these inhibitors that we are still writing the article. The information gathered in the binding process has covered the results of the unbinding process. Also there some valuable research regarding only unbinding pathways investigation such as: DOI: 10.1038/srep11539 , DOI: 10.1126/sciadv.1700014, DOI: 10.1073/pnas.1424461112.

**Comment #7:**

Provide a figure showing the unbinding along with the simulation time.

**Answer #7:**

In the Table of Contents graphic section, we provided a schematic picture of one of the compound 9 and 10 unbinding pathways as an example. RMSD values of the ligand from binding to complete unbinding positions are also available ( Fig4 a,b). RMSD plots show how ligand location changes with time as compared to their crystallographic binding poses.

**Comment #8:**

Provide pull parameters in the methods section. Also, briefly explain the general theory of Supervised Molecular Dynamics. How the molecules are pulled out of the pocket, in what direction were the ligands pulled, etc.

**Answer #8:**

We use a simulation method (SuMD) entirely different from “umbrella sampling” to pull ligands from their binding sites. A brief explanation of the SuMD method is available on lines 91-97. The directions of inhibitor unbinding are available in the Result and Discussion section, for example: " In rep2 and 3, the inhibitor unbounded between semi-open flaps forms (Figure 14e, 14f) (Figure 15a, 15b, 15c)".

**Comment #9:**

Define abbreviations in the Abstract section.

**Answer #9:**

The requested correction was done in the Abstract section (line 25).

**Comment #10:**

Why OPLS all-atom force field was used?

**Answer #10:**

Because we used the OPLS force field for binding simulations, the unbinding simulations were also done by it to reproduce the data. Also, in some other similar research involving: <https://doi.org/10.1093/bioinformatics/btaa565>, DOI: 10.1126/sciadv.1700014, this force field is used. In OPLS, surfaces (parameters) are fit to experimental data sets MM PES, so this FF is perfect for describing the properties of small molecules in their condensed states. OPLS-AA is entirely open-source, so it can be applied in many MD programs; particularly, the most popular MD program has already integrated the OPLS-AA into it.

**Comment #11:**

Authors have not provided the data of external pulling force and contacts to show the unbinding pathway of the selected molecules.

**Answer #11:**

The process of unbinding from binding pose to the full unbind is dependent on a set of factors, including aspects of protein-ligand interactions, water mediation, protein, and ligand fluctuation. In the Result and Discussion section, we explained the atomistic details of all of these factors with different analyses. As we answered in “Comment#8” we did not use pull parameters.

**Comment #12:**

Results and discussion lack data and need to be elaborated in comparison with other computational data based on similar studies.

**Answer #12:**

In the other computational research, the study's goal differed. Some of our data were new, so they could not be compared, except our other team's research that works on unbinding pathways of Indinavir in complex with HTLV-1 protease. Previously, we discussed our correlation of results in the conclusion section "These residues' interactions are unfavorable to Indinivar's stability in complex with HTLV-1 protease and result in it being a weak inhibitor".

**Comment #13:**

Authors have advised checking the quality of the minimized structure of the 1HTLV-1 and the authors should carry out additional docking studies with experimentally known inhibitors and compare the computational inhibition values with the experimental values. Without any experimental support or validation studies, the in silico binding free energy calculations may lead us totally wrong results, and the whole work may be nonsense.

**Answer #13:**

In the other project, we investigated the binding pathways of the two inhibitors, which will be available soon. By combining the results of this article and binding study results, we can have a more positive attitude towards drug design.

Only crystallographic binding pose information is analyzed in the experimental research, but we study the interactions during the pathways. As you run more simulations, you will discover more pathways. However, we added the results compared with experimental research in the conclusion section (lines 433-434 and 460-467) to better understand.

**Few minor comments;**

We had two mp forms of the potent compound (AspH32 and AspH32′) like the weak compound. In this regard, in the duration times of 4.4us" Make corrections to the unit of time.

What is mp state? The authors have advised to provide abbreviations at their first use.

"In the weak inhibitor unbinding pathways, Trp98 and Trp98′ with pi-pi interactions, due to their close position to one of the exit areas were not good supporters for Asp32 or Asp32′, like His66′, His66′," Author should correct the notation of His residue.

Authors have calculated the total interaction energy of compound 9 and 10 with htlv but did not mention anything about it in the text. Merely displaying in figures does not make sense.

"We had two mp forms of the potent compound (AspH32 and AspH32′) like the weak compound." Should mention properly which is potent and each compound.

Authors already mentioned that one compound is better than other and they are just investigating the mechanism of unbinding. In MS authors tells about two mp form of compounds (potent and weak). Is the mp forms be taken into account for getting Ki values for these compounds?

**Answer:**

A unit correction was done. The abbreviation of mp was added in the Abstract section. The correction was done in "In the weak inhibitor unbinding pathways, Trp98 and Trp98′ with pi-pi interactions, due to their close position to one of the exit areas were not good supporters for Asp32 or Asp32′, like His66′".

Total interaction energies plots are obtained from the sum of the coulomb and Lennard-jones contribution values. We have maximum interaction energies in the bound state of the protein-ligand complex. As time passes and protein-ligand interactions weaken, the values of the plot increase and eventually reach zero until full unbinding. So all the interactions energies plots' trend is correlated with the unbinding process from the bound state to the unbound state. So we used these plots to prove full unbind occurrence by energy values.

Aspartic proteases tend to cleave substrate by monoprotonation of aspartates in the catalytic dyad. Since HIV protease is an aspartic protease, and unfortunately, there was no typical study about HTLV-1 protease, we decided to take a pattern from HIV research. So in some research, they do not apply protonation or do not mention it, or in some research, they apply protonation state in chain A. Due to we wanted to have more complete outcome, we decided to use protonation in both chains separately.

We mentioned in the conclusion section, "Herein, we cannot conclude certainly which state of protonation actually occurs". In the presence of an inhibitor, the kind of protonation that will occur can only be determined when exposed to the active site. Because of this, we decided to discuss protonation more cautiously and, instead of judging, report the results.

**Reviewer #2:**

**Comment #1:**

What purpose do authors have selected for two different structures of HTLV-1 protease for this study?

**Answer #1:**

As we mentioned in the Result and Discussion section, "Since the only structural difference between compounds 9 and 10 is in the amino and nitro groups on the benzene ring (Figure 1a, 1b), compound 10 (K_i_ = 15 nM) is approximately 526 times more potent in complex with HTLV-1 protease. Therefore, a proper understanding of the unbinding pathways of these compounds is vital to unveiling secrets that a minor structural difference can have a dramatic effect on inhibitory effects." We could monitor critical factors from a comparative perspective, which made the results more reliable for designing new inhibitors. The related section improved in the manuscript.

**Comment #2:**

The authors have used co-crystallized ligands in this study. What is the novelty of the proposed study?

**Answer #2:**

There is no FDA-approved drug for this target, so we decided to work on designed inhibitors. In our view, it is critical to determine the negatives and the positives of the current potent inhibitor. We also compared the result with a weak inhibitor to achieve a complete insight for designing next-generation inhibitors.

**Comment #3:**

From the literature, it was reported that the amino acid Met37 played a key role in the binding of inhibitors in the active site of HTLV-1 protease. Did authors have noticed interaction with this amino acid in this study?

**Answer #3:**

In our replicas, Met37 effects were not significant as critical residues. Important interaction energies values are available in energy plots.

**Comment #4:**

Authors have mentioned the uniform mechanism to unbind with some important differences: what are key features or differences noticed during the simulation period?

**Answer #4:**

The Phrase "we saw a uniform mechanism to unbind with some important differences that caused a significant difference in one of simulation time" was used for compound 10 unbinding pathways in complex with HTLV-1 protease when Asp32 of chain A was protonated. We mentioned "In this state, Lys95′ by forming a hydrogen bond with the atom of O_10_, along with His66′ cation-pi interaction with the benzene ring, was a third essential residue" (lines 231-232) and "According to significant differences in replicas simulation times, the effects of the Lys95′ hydrogen bond (Figure 4i) appear more pronounced" (lines 235-236) as a key factor that caused significant different in simulation time in the Result and Discussion section.

**Comment #5:**

Authors have mentioned that the compound shows 526 times more potent in complexes with HTLV-1 than compound 9, is the substitution of the amino group of this compound enhances the activity of compound 10?

**Answer #5:**

Yes, it is. The inhibitor 10 could be able to interact with His66′ and Lys95′, which are essential.

**Comment #6:**

Does the amino group of the compound 10 show any interaction with the important amino acid residues of the HTLV-1 protease?

**Answer #6:**

Yes, it does. Asp36′ by forming H-bond when Asp32 of chain A was protonated, and Asp36 and Asp36′ by forming H-bonds when Asp32 of chain B was protonated with the amino group, which is available in the text line 226 and line 270-274.

**Comment #7:**

Authors have mentioned that the positive charge of the pyrrolidine ring plays a crucial role in preserving ligands. Both compound 9 and 10 have pyrrolidine rings in their structure, then how compound 9 shows weaker activity than compound 10? Does any specific mechanism play a role in activity of compound 10?

**Answer #7:**

We mentioned in the Conclusion section, "The pyrrolidine fragment was held well by Asp32 or Asp32′ from the native binding pose of the two compounds. Thus the interactions of other fragments with other residues in different protein regions caused significant differences." Moreover, "His66′ with its cation–pi interaction with an aniline ring of inhibitor was a perfect supporter to Asp32′. This residue's effect was absent in the other form of protonation state and caused a significant difference in simulation time. In the weak inhibitor unbinding pathways, Trp98 and Trp98′ with pi-pi interactions, due to their close position to one of the exit areas, were not good supporters for Asp32 or Asp32′, like His66′. His66′, due to far position from the bottom and center of the binding pocket, could fixed aniline fragment and decreased ligand fluctuations. The two mentioned tryptophan were closer to the important aspartic acids, and there was enough space for ligand fluctuations. For this reason, Asp36 in the active site that was close to the exit area could be a competitor with Asp32′ and was not a good interaction for keeping the ligand in the binding pocket. Similarly, attenuating effect of Trp98/Trp98′ residues in unbinding pathways of the weak inhibitor correlates with another research result" <https://doi.org/10.1371/journal.pone.0257916>

**Comment #8:**

In the main text it was mentioned that the figure 4 c-e was the first state of rep1, 2, and 3, but in figure it was mentioned as ligand RMSD. Make it correct.

**Answer #8:**

In Figure 4 , ligand RMSD values are for 4a and 4b.

**Comment #9:**

In figure 5b and c, both lines were depicted as Rep2 and Rep3-aniline-benzene. Then how does it show the difference in the distance? What are the main differences noticed in these structures?

**Answer #9:**

These plots aim to show and prove pi-pi intramolecular interactions in the inhibitors. So we considered the two aniline-benzene rings in compound 10 and the two nitrobenzene-benzene rings in compound 9, which were closer together to make intramolecular interactions. These plots should not compare with each other.

**Comment #10:**

Is the author using compound 9 as the weaker inhibitor in this study?

**Answer #10:**

Yes

**Reviewer #3:**

**Comment #1:**

After lots of simulations and analyses, it is still unclear that which states of protonation plays the deciding role there. It would be better if the authors are critical on this point because it is one of the most important viewpoints of this work.

**Answer #1:**

According to all forms of protonation, there are 16 forms. A QM/MM study will help us determine which protonation forms are closer to the natural form. Also, Aspartic proteases tend to cleave substrate by monoprotonation of aspartates in the catalytic dyad. Since HIV protease is an aspartic protease, and unfortunately, there was no typical study about HTLV-1 protease, we decided to take a pattern from HIV research. So in some research, they do not apply protonation or do not mention it, or in some research, they apply protonation state in chain A. Due to we wanted to have a complete outcome, we decided to use protonation in both chains separately.

**Comment #2:**

Authors in many places discussed about pi-cation and pi-anion interaction (e.g., His66 interacts with aniline in cation-pi interaction, Asp36 forming pi-anion interaction with nitrobenzene etc.). It is important to note that the energetics of such interactions are very important. (For example, pi-anion interactions have energetics in the range around 20 to 50 kj/mol whereas pi-cation interactions have energetics in the range of -10 to -20 kj/mol). The authors should check the energetics of such interactions here or at least cite relevant papers where people have reported the energetics of these particular system.

**Answer #2:**

We first reported the interaction energy values, so we cannot compare them or cite other works. We chose significant interactions based on the average amount of most important interaction energies of the protein-ligand complex. Our study values are negative and favorable since the nitro group is a strong electron acceptor and positively attaches to the ring. It can easily be made by pi-anion interaction with aspartic acid, which carries a negative charge.

**Comment #3:**

Authors should rigorously work on the figure qualities. Those are not good at publication format presently. For example, Figure 13, c, d, e. Note that, the labellings are very bold and not as per the mark of publication. Also, in 13a, the x-axis labellings are very closely spaced (10,20,30,40….80) makes it looks worse. They should scrutinize the figure qualities and font labelling throughout in-depth.

**Answer #3:**

The requested changes were applied to the figures.

**Comment #4:**

In Figure 10 caption (where they emphasize the details of compound 9 unbinding pathway), just before the last line, they mentioned "k, l, 3", What is "3" here? I did not find anything such and believe "3" should be replace by "m". Such things they should check thoroughly.

**Answer #4:**

The corrections were done.

**Comment #5:**

It is better to follow a uniform free energy bar throughout which is currently missing. For example, Figure 7, c, d, e. Their energy bars are not uniform (For c, it is 0 to 16.90 and it is different for d and e.). The authors should follow uniform energy bar for all, otherwise the comparison is not at all logical.

**Answer #5:**

The FEL plots were prepared by python, and the python codes made the energy bars automatically from different values obtained using "gmx sham" files in different replicas. Also, we tried uniforming plots scale, but some of the plots became too compact and became difficult to separate steps.

**Comment #6:**

The authors mention that "For our purpose, we had various short and long-time-scale simulations". I understand they chose based on the unbinding time scales, still did the authors performed any convergence tests or something like that, to choose time scales in this study?

**Answer #6:**

We did not do that. Because all full unbound occurred automatically without applying any biasing force. Various simulation times showed various unbinding processes. We corrected the text to prevent misinterpretation ( line 208).

**Comment #7:**

Figure 5, for the three replica's they chose three different time scales, that should be addressed properly. What happened if for Figure 5b and 5c, they did 4000 ns simulations like Figure 5a? Then, how the distance plot would look like? Since, they are doing unbiased simulations, these questions need to be answered. Also, it is visually more sound to have similar time-scales for different replica's.

**Answer #7:**

The time it takes for an inhibitor to leave its binding pocket depends on the pathway it is sampled. Unbinding pathways may contain multiple energy barriers, so the residence time of the inhibitor at the binding site increases, and it leaves the protein later. The inhibitor or replica whose unbinding pathway has fewer energy barriers exits the protein faster. All replicas are simulated under identical conditions using SuMD.

**Comment #8:**

Figure 3g, take care of the x axis label. It has been cut-off (120 ns is cut-off there). Same goes for Figure 8c. Also, The TOC (as well as TOC fonts) is not looking good as per the journal standard.

**Answer #8:**

The correction was done, and also revised version of the article was changed to meet PLOS ONE's style requirements.

**Comment #9:**

The article is somewhat looks to be less referenced. For an example, they should cite some important references regarding the interactions such as the review articles sastry et al doi: 10.1021/cr300222d and doi: 10.1021/jp900013e, and some others, for example <https://doi.org/10.1021/ct200569x>,<https://doi.org/10.1021/acs.jpcb.7b01736>and <https://doi.org/10.1021/acs.jpcb.9b06343> etc.

**Answer #9:**

Some references were added in lines 224 and 340.
