# Supplementary material for: Comparative study of the unbinding process of some HTLV-1 protease inhibitors using unbiased molecular dynamics simulations
Source: PLoS One. 2022 Jul 14;17(7):e0263200. doi: 10.1371/journal.pone.0263200 (PMC9282663; doi:10.1371/journal.pone.0263200)
Supplement: Attachment — Submitted filename: Plos_review.docx [file pone.0263200.s005.docx]

The author addresses most of the things to make the quality of the paper better yet a few issues should be addressed before further consideration.

- The author mentioned to my first query that “So in some research, they do not apply protonation or do not mention it, or in some research, they apply protonation state in chain A” …. It would be good if they discuss those studies briefly in the revised manuscript to get the reader a subtle grasp about the history of the study which is still somewhat missing.
- According to my comment 5 regarding uniform free energy bar…. I think it’s needed (Even if not in the manuscript, then in the SI) because that not only establish the fidelity of the work but also required for proper comparison between the states. This is because, we know the free energy changes dramatically based on scaling and non-uniformity of the values selected.
- I am somewhat surprised regarding the revision of the author of my comment 9. They did not incorporate none of the suggested studies and just incorporated two different references. Those suggested literature evidence are important and need to be incorporated.
