# Supplementary material for: Comparative study of the unbinding process of some HTLV-1 protease inhibitors using unbiased molecular dynamics simulations
Source: PLoS One. 2022 Jul 14;17(7):e0263200. doi: 10.1371/journal.pone.0263200 (PMC9282663; doi:10.1371/journal.pone.0263200)
Supplement: Attachment — Submitted filename: Response to Reviewers.docx [file pone.0263200.s006.docx]

**Dear Prof. Sandipan Chakraborty;**

I am pleased to mail you the second revision of the article titled **"Comparative study of the unbinding process of some HTLV-1 protease inhibitors using Unbiased Molecular Dynamics simulations"**. All comments have been answered upon reviewers' recommendations, and the corrections were made in manuscript #PONE-D-22-00753R1. It is our pleasure to hear your feedback and any other suggestions relevant to the paper.

**Sincerely,**

**Hassan Aryapour**

**Comment #1:**

The author mentioned to my first query that “So in some research, they do not apply protonation or do not mention it, or in some research, they apply protonation state in chain A” …. It would be good if they discuss those studies briefly in the revised manuscript to get the reader a subtle grasp about the history of the study which is still somewhat missing.

**Answer #1:**

The requested description was added along with the reference (lines 113-117)

**Comment #2:**

According to my comment 5 regarding uniform free energy bar…. I think it’s needed (Even if not in the manuscript, then in the SI) because that not only establish the fidelity of the work but also required for proper comparison between the states. This is because, we know the free energy changes dramatically based on scaling and non-uniformity of the values selected.

**Answer #2:**

Yes, it is definitely easier to compare between states if the maximum values on the x and y scale are the same in all graphs. So once again, according to your request, we considered the maximum values of the x and y axes to be the same. Unfortunately, since in some replicas, the amount of maxima is significantly different from each other, a lot of space on the plots becomes empty and white, so the maximum amount of axes is automatically adjusted based on the maximum amount of data after running the script. However, the changes you requested were added to the supplementary section.

**Comment #3:**

I am somewhat surprised regarding the revision of the author of my comment 9. They did not incorporate none of the suggested studies and just incorporated two different references. Those suggested literature evidence are important and need to be incorporated.

**Answer #3:**

All the requested references were added (refs : 18, 38,39).
