# Supplementary material for: Comparative study of the unbinding process of some HTLV-1 protease inhibitors using unbiased molecular dynamics simulations
Source: PLoS One. 2022 Jul 14;17(7):e0263200. doi: 10.1371/journal.pone.0263200 (PMC9282663; doi:10.1371/journal.pone.0263200)
Supplement: Attachment — Submitted filename: Response to Reviewers.docx [file pone.0263200.s007.docx]

**Dear Prof. Sandipan Chakraborty;**

I am pleased to mail you the second revision of the article titled **"Comparative study of the unbinding process of some HTLV-1 protease inhibitors using Unbiased Molecular Dynamics simulations"**. All comments have been answered upon reviewers' recommendations, and the corrections were made in manuscript #PONE-D-22-00753R1. It is our pleasure to hear your feedback and any other suggestions relevant to the paper.

**Sincerely,**

**Hassan Aryapour**

**Comment:**

Authors successfully address the reviewer concerns. However, the following issue needs to be clarified before any final decision on the manuscript.

The author of the manuscript entitled "Comparative study of the unbinding process of some HTLV-1 protease inhibitors using Unbiased Molecular Dynamics simulations" performed ligand unbinding from the protein HTLV-1. They consider one high affinity ligand and a low affinity ligand. However, the author published a previous paper on "Comparative analysis of the unbinding pathways of antiviral drug Indinavir from HIV and HTLV1 proteases by supervised molecular dynamics simulation" where they consider unbinding of another ligand Indinavir. The method is very similar and presentation is also very similar. A small discussion is there in the manuscript. But the author needs to justify their objective to work on different ligands and clearly define the novelty of the work in light of previous publication.

**Answer:**

We said in lines 59-69 that many research groups had designed inhibitors, but the FDA has approved none. For this reason, if we want to design successful inhibitors, we must examine existing inhibitors' negative and positive points. These will ultimately lead to valuable information for drug design.

In a previous project, our research team discovered the reasons for Indinavir's ineffectiveness against HTLV-1 protease. As we continue, we examined the unbinding pathways of two inhibitors (the most potent and one of the weakest) in complex with HTLV-1 protease. Although the study method was the same, the aim of the research was different since, in the previous project, we had one drug and two different target proteins, while in the present study, we have two different inhibitors for only one target protein.

More information was added in the Introduction section (lines 98-100), and the common results of these two projects were mentioned before in lines 456-457.
